# Evaluation of the effects of hydrogel and glycine betaine application on growth, physiological characteristics and yield of peanut under water deficit

**DOI:** 10.1186/s12870-026-08679-4

**Published:** 2026-04-16

**Authors:** B. A. Bakry, M. E. Nowar, M. E. El-Awadi, G. Sh. Bakhoum, M. S. Sadak

**Affiliations:** 1 Field Crops Research Department, Agricultural and Biological Research Institute, National Research Centre, P.O. 12622, 33 El Bohouth Street , Giza, Dokki Egypt; 2 Botany Department, Agricultural and Biological Research Institute, National Research Centre, P.O. 12622, 33 El Bohouth Street , Giza, Dokki Egypt

**Keywords:** Glycinebetaine, Hydrogel, IAA, Oil, Osmoprotectants, Peanut, Protein, Water deficit

## Abstract

The main objective of all countries, particularly those in dry regions, is to preserve water resources. This study addresses the knowledge gap regarding effective strategies to mitigate drought-induced damage and enhance productivity in peanut plant. Accordingly, during two summer seasons of 2023 and 2024, a field trial was conducted to investigate the impact of hydrogel addition to sandy soil and foliar spraying of glycinebetaine (GB) on physiological parameters, growth, yield, its components, and nutrients of peanut seeds subjected to water deficit stress. Water deficit (75% of the water irrigation quantity, WIQ) resulted in reduced levels of chlorophyll a, chlorophyll b, carotenoids, indole acetic acid (IAA), growth, seed yield, oil, carbohydrates, and protein in the yielded seeds compared to normal irrigation (100% WIQ). However, hydrogel treatment or GB treatment alone or in combination showed a stimulatory effect on growth criteria and yield attributes of peanut plants under 100% WIQ and 75% WIQ via improving chlorophyll a, chlorophyll b, carotenoids, IAA, total soluble sugars and phenols. Hydrogel soil addition with GB foliar treatment exhibits more increases in the above-mentioned parameters over hydrogel or GB treatment alone under 100% and 75% WIQ. Additionally, 20 mM GB with hydrogel 80 kg ha^-1^ worked better over other treatments, as it caused the highest values of increases in most studied parameters. It increased seed yield (ton ha^-1^) and biological yield (ton ha^-1^) under 100% WIQ by 72.45% and 78.92% and under 75% WIQ by 141.37% and 112.97% respectively. These results show that GB, when combined with hydrogel polymer, significantly improve drought tolerance in peanut, providing a viable strategy to increase crop yields in water-limited environments. In view of climate change, this study emphasizes the potential of combining hydrogel with GB for sustainable agricultural practices.

## Introduction

Water deficit is one of the major challenges that is limiting plant growth and production worldwide, and as climate change and global warming intensify the problem, they pose a serious threat to global food security, which is a major problem to food security [1]. Drought is a powerful abiotic stressor that halts crop development, greatly impacting crop health and yields [2, 3]. Drylands, covering nearly 41% of the world’s land area, are marked by scarce rainfall and elevated evapotranspiration rates. These severe conditions result in considerable moisture loss [4, 5]. Given the current state of climate change, both the duration and frequency of water deficit periods will increase in the upcoming years, making this one of the century’s most significant concerns [6], they pose a serious threat to global food security [7]. More food shortages and devastating droughts will occur in African countries. Many people will be at risk and food insecurity will rise if low-income nations in Asia or Africa see a drop in agricultural output as a result of climate change [8]. Water deficit significantly lowered plant growth and production, and it negatively impacted a number of physiological activities, including respiration, photosynthesis, ion uptake and translocation, nutrient metabolism, carbohydrate metabolism, and chlorophyll biosynthesis [9, 10]. Furthermore, lipids, proteins, and nucleic acids are damaged when reactive oxygen species (ROS) formation and scavenging are out of equilibrium, which can occasionally result in plant cell death [6, 11]. Drought stress is directly linked to factors that reduce plant growth and crop potential [12–15]. In response to drought, plants increase the manufacturing of compounds that provide protection [16]. These include: (a) osmolytes (soluble carbohydrates, amino acids, amines, and polyols) that function as both compatible solutes, stabilising cellular proteins and structures, and osmoprotectans, preserving cell turgor under osmotic stress caused by drought (b) substances and mechanisms that aid in the dissipation of thermal energy and upholding redox regulation [17].

Innovative approaches are being investigated to improve crop resistance to water-limited circumstances and increase water use efficiency in response to these problems. Hydrogels, which are extremely absorbent polymers that can hold a lot of water and release it gradually over time, are one such alternative. The potential of hydrogels to enhance soil moisture retention, lessen water loss by evaporation and deep percolation, and promote plant development during dry spells has drawn interest [18]. Furthermore, ideal hydrogel materials should be non-toxic, highly absorbent, cost-effective, durable, stable, photo-stable, neutralize their pH after swelling in water, and biodegradable without producing toxic substances [19]. According to earlier studies, hydrogels can improve the physical (porosity, bulk density, water-holding capacity, soil permeability, and infiltration rate), chemical and biological properties of soil, especially in dry and semi-arid areas [17]. Since the improved soil porosity resulted in enhancement of root growth and density, seed germination, and the rate at which seedlings emerged, as well as decreased soil erosion because of less compaction [20]. Hydrogels have been shown to enhance plant water availability in sandy soil by reducing soil hydraulic conductivity, increasing retention pores, and reducing drainage loss [21]. It’s crucial to remember that HPMC-Xanthan gum hydrogel can be used as a practical technique to reduce the quantity of water required for soybean plant irrigation, as mentioned by Elsayed [22].

Another commonly used tactic is exogenous administration of glycinebetaine (GB). Chloroplasts naturally produce GB, one of the quaternary ammonium compounds known as osmolytes, in response to abiotic stressors such salt and water deficit [23–25]. GB protects membrane integrity, enzyme and protein complex functioning, and cell structures from the damaging effects of water deficit, in addition to its osmoregulatory role [26]. By stabilizing proteins and enzymes, detoxifying reactive oxygen species, aiding in cellular osmotic adjustment, and boosting the activity of antioxidative enzymes, GB generally helps plants deal with stress in a number of ways [27, 28]. Exogenous GB spraying has been shown to stabilize the net photosynthetic rate, photosynthetic pigments, and chlorophyll fluorescence. Additionally, it can boost the amount of chlorophyll, promote plant development, and produce more leaves [29]. GB also improves plant tolerance by decreasing lipid membrane breakdown and stopping photo-inhibition [30]. GB restores crop growth and seedling development in the event of a water deficiency [31]. It has been shown that exogenous GB therapy is a practical way to directly maintain and enhance maize growth and productivity [32]. Islam [33] claims that GB foliar spray enhanced the overall performance of the oilseed crop. Given that mustard growth is positively impacted by a 20 mM GB foliar spray.

Peanuts (*Arachis hypogaea* L.), often known as groundnuts, are one of the most important summer oilseeds and proteins. They are the king of oilseed crops and edible legume seeds [34]. Peanuts are the twelfth food crop, the fourth oilseed crop, and the third source of vegetable protein. In Egypt, peanuts have been grown on an average of 62,000 hectares of land over the last five years [35]. Peanuts are as popular as they are nutrient-dense, containing 40–50% oil, 25–30% protein, 20% carbohydrates, and 5% ash, in addition to additional minerals including calcium and magnesium, depending on the variety and growing methods [36]. In Egypt, peanuts are not used to obtain oil but to get cash by exporting them. So, farmers considered it a great cash crop, especially in newly reclaimed soil. Additionally, peanuts’ short soil lifespan allows for larger economic returns in recently reclaimed lands than other crops, which is primarily responsible for their economic benefits. Peanut grows up in Egypt in sandy soils during the summer season, which is characterized by high temperature and the presence of water deficit problems and increasing evapotranspiration with low water holding capacity [37]. Additionally, peanut leaves are fed to animals. Thus, this crop has drawn the attention of both the government and experts due to its capacity to thrive in recently recovered sandy soil. Peanuts are used not just to produce oil but also for peanut butter, candy, roasted peanuts, snack foods, meat product extenders, soups, and desserts [38].

This work fills in the information gap about practical methods for reducing damage caused by drought and increasing peanut plant output. We postulated that by altering the peanut’s physiological and biochemical responses, hydrogel and GB treatments could increase drought tolerance. Even with these developments, there is still much to learn about the use of hydrogel and GB to reduce water deficiency stress in peanut plants. According to earlier research, applying hydrogel or GB to several plants under water deprivation stress situations offers encouraging outcomes. Nevertheless, it is still unclear how hydrogel and GB interact with plants. In order to mitigate the lessened effects of drought, this study emphasizes the physiological role of hydrogel and GB exogenous treatment on peanuts.

## Materials and methods

### Experimental procedures

The experiment was carried out to study the effect of hydrogel soil applications at different levels (0, 40 and 80 kg ha^− 1^) and foliar application of glycinebetaine (GB) at rates of 0, 10 and 20 mM under two water irrigation levels at normal irrigation (100%WIQ) and water deficit stress (75% WIQ), on growth, yield and its components, as well as seed quality of peanuts under sandy soil conditions. A field experiment was carried out at the Experimental Station of the National Research Centre, Al-Nubaria district, El-Beheira Governorate, Egypt, during summer months of 2023 and 2024 seasons. The site is 21 m above sea level and is situated in latitude 30°30′1.4′′N and longitude 30°19′10.9′′E.

Prior to peanut planting, soil samples were collected for soil analysis at two different depths: 0–30 cm and 30–60 cm below the soil surface [39]. (Table 1) displays the results of chemical and physical (texture) analysis of the soil at the experimental site.

**Table 1:** Physical (Texture) and chemical analysis of the experimental soil before sowing

Physical (Texture) analysis of the experimental soil						
Season	Constant depth (cm)	Coarse sand (%)	Fine sand (%)	Silt (%)	Clay (%)	Texture class
2023	00–30	39.3	44.5	10.9	5.3	Sandy
	30–60	39.5	44.4	10.8	5.3	Sandy
2024	00–30	38.6	42.9	12.7	5.8	Sandy
	30–60	38.6	41.3	13.5	6.6	Sandy

Chemical Analysis of the experimental soil													
Season	Constant depth (cm)	pH	EC (dS/m)	Sat (%)	Anions (meq/L)			Cations (meq/L)				CaCO_3%_	OM%
					HCO_3_^−^	Cl^−^	SO_4_^−2^	Ca^2+^	Mg^2+^	Na^+^	K^+^		
2023	00–30	7.87	1.26	32	0.58	8.7	1.28	1.9	1.2	7.16	0.3	1	0.40
	30–60	7.88	1.79	28	0.61	8.3	1.46	2.17	1.5	6.3	0.3	6	0.37
2024	00–30	7.98	1.59	24	0.31	12.8	1.9	2.8	2.2	9.8	0.3	1.9	0.38
	30–60	8.00	1.81	24	0.4	14.4	2	4.2	2.4	9.9	0.3	1.3	0.32

Variety Gize-5 of the peanut certified seeds (*Arachis hypogaea* L.) was obtained from the Oil Crops Research Section of the Field Crops Research Institute of the Agricultural Research Center in Giza, Egypt. Giza-5 variety has the highest protein content and thrives in newly reclaimed lands. It was inoculated with the appropriate rhizobium bacteria inoculants shortly before planting, in both seasons, peanut seeds were sown in the first week of May. The experimental soil was ploughed twice. A boarder of 1 m was left between each two experimental main plots to avoid irrigation effects. Peanut was sown by drilling peanut seed at a distance of 10 cm apart in the assigned ridges on one side of the ridge. After the germination was completed, peanut seedlings were thinned to one plant per hill. During the preparation of the seed bed, phosphorus fertilizer in the form of calcium superphosphate (15.5% P_2_O_5_) was supplied at rate of 145 kg P_2_O_5_ ha^− 1^. At sowing, 120 kg ha^− 1^ of potassium sulphate (48% K_2_O) was used. At a rate of 72 kg N ha^− 1^, ammonium sulphate (20.6% N), nitrogen fertilizer was supplied in two equal parts: the first half at planting and the second after 30 days after sowing. The experiment was designed in split-split plot system of the randomized complete block design (RCBD) and three replications was used, where water irrigation quantities (WIQ) 100% and 75%,WIQ were located in main plots, where in hydrogel soil applications at three levels of (0, 40 and 80 kg ha^− 1^) were randomly distributed in sub plots and the GB at rates of **(**0, 10 mM, 20 mM) were allocated in sub-sub plots and carried out two times as foliar application at the beginning of budding (30 days from sowing) and flowering (45 days from sowing). The used concentrations were chosen according to a preliminary germination experiment using different concentrations of GB (0.0, 5, 10, 15, 20, 25 and 30 mM), then, the appropriate concentrations were chosen based on the results of growth characteristics of this experiment. GB was freshly used after being dissolved in distilled water at the selected concentrations. All spray treatments were completed early morning, before 9:00 a.m., with a hand sprayer at sufficient pressure to keep droplet size small. To obtain proper coverage, plants were sprayed from all sides. The spray volume amount of water consisted of approximately 500 l ha^− 1^. Plants were sprayed from both sides of the row to achieve adequate coverage. The plot area was 10.5 m^2^ consisting of five rows (3.5 m length and 60 cm between rows). Glycinebetaine was obtained from Sigma-Aldrich Company; Hydrogels are polymer materials having a three-dimensional (3D) network. These materials are formed synthetically from natural sources which are stable and hydrophilic in nature. hydrogel as dry granules were mixed directly into the soil in the opened furrows before sowing then, covered and sowed by peanut seeds. However, the main properties of the used superabsorbent hydrogels are presented in (Table 2).

**Table 2:** Physio-chemical contents of the used hydrogel. This the commercial product imported from agrofrance international company, France by Elbarbary plant company in Egypt

Parameters	Characteristics
Chemical constituents	Cellulose-based grafted cross-linked anionic polyacrylate
Appearance	Amorphous, granulous
Particle size	20–100 mesh (micro-granules)
PH	7-7.5
Stability at 50̊ C	stable
The least deionized water absorption rate	350 gg^− 1^
UV light sensitivity	None
Temperature for maximum absorption	50 °C
Required time for 60% swelling	2 h (approx.)
Stability period in soil	< 2 years
Toxicity in soil	None

### Water irrigation requirements

Water irrigation requirement was determined according to Allen [40]. The growing peanut plants were irrigated every two days. The average amount of irrigation water applied with the sprinkler irrigation system was 4760 and 3750 m^3^ ha^− 1^ season^− 1^ (for 100% and 75%) for the two seasons [41]. The following equation was used to determine the irrigation water amounts:

### Water productivity (WP)

The WP calculation was carried out as demonstrated by Howell [42], who acknowledged the connection between seed yield and irrigation water quantity. The following formula was used to calculate WP in kg mm^3^ ha^1^: WP = Ey / Et.

Where WP is the water productivity (kg/m^3^); Ey is the economical yield (kg ha^− 1^); and Et is the total irrigation water used, m^3^ ha^− 1^ season.

### Biochemical estimation

After 60 days of sowing, five plants were selected from each plot to measure biochemical estimations:

Chlorophyll a, chlorophyll b and carotenoids were determined in leaf tissue by Lichtenthaler and Buschmann [43]. Indole acetic acid content (IAA) in leaf tissue was determined according Gusmiaty [44]. Total phenol in leaf tissue was determined by Gonzalez [45]. Proline content in leaf tissue was measured using the technique indicated by Verslues, [46]. Free amino acids in leaf tissue were measured according to Sorrequieta [47].

#### Seed chemical analysis

Oil content of seeds was measured according to Das [48]. Total carbohydrate content in peanut seeds was measured in accordance with Albalasmeh [49]. Total protein in seeds was calculated in accordance with the procedure published by Latimer [50].

### Growth parameters

After 60 days of sowing, five plants were selected from each plot to measure morphological parameters like shoot length (cm), number of branches and leaves per plant, shoot fresh and dry weight (g) per plant, and root length (cm) and fresh and dry weight (g) per plant.

### At harvest yield measurements

At harvest time, a sample of five plants was collected from each plot, data on seed yield characters were recorded as follows: Plant height (g), number of pods/plant, pod yield /plant (g), seed yield/plant (g) and 100 seeds weight (g). Plants of the whole plot were harvested and the pods were removed to calculate: straw yield (ton/ha.), biological yield (ton/ha.), pod yield (ton/ha.), seed yield (ton / ha.), oil yield (kg/ha.) and protein yield (kg/ha.).

### Statistical analysis

Data were subjected to the analysis of variance for split-split plot design using SAS Statistical Software, Package v.9.2 [51]. Given that the tendency was similar between the two seasons, the homogeneity test using Bartlet’s equation aided in integrating the analyses of the two seasons and treatments means were compared using Duncan (1955) test at 5% of probability [52]. The correlation coefficients among studied traits for both growing seasons were computed using the Genstat Prog. software version 20th edition according to Payne [53].

## Results

### Changes in photosynthetic contents

**Fig. 1:**
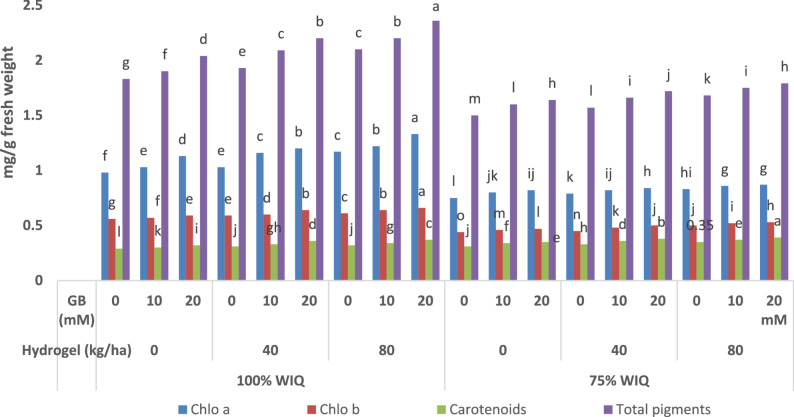
Mean values within the same column for each trait with the same lower-case letter are not significantly different at p ≤ 0.05 Impact of water irrigation quantity, hydrogel and GB and their interaction on photosynthetic pigments (mg/g fresh weight) of peanut plants

Undoubtedly, the well-watered (100% WIQ) peanut plants have the highest significant *p* ≤ 0.05 photosynthetic pigment (chlorophyll a Chlo a, chlorophyll b Chlo b and total pigments) contents while decreasing significantly carotenoids as compared to the water –deficit- stressed (75% WIQ) plants (Fig. 1). Moreover, hydrogel polymer soil amendment with two levels (40 and 80 kg ha^-1^) significantly enhanced Chlo a, Chlo b, carotenoid and consequently total photosynthetic pigments, as compared with untreated soil treatment. Also, spraying GB with 10 and 20 mM significantly enhanced the photosynthetic pigment attribute values as compared with untreated control. Furthermore, hydrogel or GB with different concentrations could alleviate partially the reduced impact of water deficit stress (75% WIQ) as hydrogel and GB increased gradually and significantly the studied photosynthetic pigments comparing with untreated stressed plants (Fig. 1). It is obvious from (Fig. 1) that, the combination of WIQ100% and 75% WIQ with hydrogel polymer (40 and 80 kg ha^-1^) and GB foliar spraying (10 and 20 mM) showed higher significant increases of chlorophyll a, chlorophyll b, carotenoids and total pigments than hydrogel addition or GB treatment alone. Hence, foliar treatment with 20 mM GB + 80 kg ha^-1^was the most effective treatment in increasing the above-mentioned parameters of peanut plants under all studied conditions (100% and 75% WIQ) (Fig. 1).

### Changes in endogenous indole acetic acid and phenols content

**Fig. 2:**
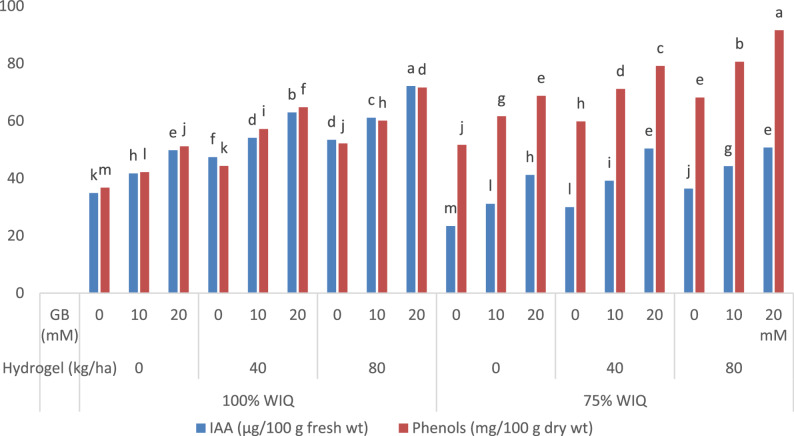
Mean values within the same column for each trait with the same lower-case letter are not significantly different at p ≤ 0.05 Impact of water irrigation quantity, hydrogel and GB and their interaction on indole acetic acid (IAA, µg/100 g fresh weight) and phenols (mg/g fresh weight) of peanut

The presented data in (Fig. 2) clearly show that, decreasing irrigation water from 100% to 75% WIQ caused significant decrease *p* ≤ 0.05 in endogenous IAA, while increased significantly phenol content of peanut leaves as compared with those plants irrigated with 100% WIQ. On the other hand, the addition of hydrogel to soil with 40 and 80 kgha^-1^ and foliar treatment with GB 10 and 20 mM caused significant increases in endogenous IAA and phenol contents of peanuts as compared with untreated control (Fig. 2). Furthermore, under drought stress 75% WIQ condition, the application of hydrogel soil amendments (40 and 80 kg ha^-1^) with GB (10 and 20 mM) significantly promoted the IAA and phenolic contents in peanut leaves as compared to the control treatment (without soil amendment and without GB). In this respect, compared to the corresponding control treatment, hydrogel polymer plus GB supply showed significant enhancements of IAA and phenolic contents. It is clear that the combination of 100% WIQ and hydrogel polymer (40, 80 kg ha^-1^) plus GB with 20 mM revealed the maximum significant contents of endogenous IAA (it increased from 34.89 to 72.12 µg/100 g fresh weight with 106.7% of increase and from 23.39 to 50.69 with 116.7% of increase under 100% and 75%, WIQ respectively) and phenol contents (it increased from 36.76 to 71.64 mg/100 g dry weight with 94.9% of increase and from 51.63 to 91.46 with 77.1% of increase under 100% and 75%, WIQ respectively) in peanut leaves compared to other treatments (Fig. 2). 

### Changes in proline and free amino acids

Regarding the performance of peanut plants in self-production of osmolytes, namely, proline, and free amino acids FAA, (Table 3) clearly shows that decreasing irrigation of peanut plants with WIQ 75% caused significant increases *p* ≤ 0.05 in proline, and free amino acid contents. The obtained data in (Table 3) clearly show that peanut plants grown in soil amended with hydrogel (40 and 80 kg ha^− 1^) and treated with GB 10 and 20 mM gave more significant increases in proline and free amino acids, compared with soil without hydrogel under both irrigation levels. With respect to the interaction effect of hydrogel addition and / or GB foliar treatment on peanut plants under different irrigation levels (normal and stressed), it is interesting to note that application hydrogel at 40 and 80 kg ha^− 1^ level or GB (10 and 20 mM) caused significant and gradual accumulation of proline and free amino acids compared with untreated control either under 100% WIQ or 75% WIQ (Table 3). Also, the obtained data obviously showed that the most effective treatment of GB was 20 mM with 80 kg/ha under 75% WIQ, it caused increases of 19.99%, 61.90, and 28.58% compared with control plants. While the increases reach 17.33%, 133.53% and 63.89% when comparing with the control plant under 100% WIQ. Additionally, both under drought stress conditions (75 WIQ) and at normal irrigation levels (100% WIQ), GB treatment plus hydrogel addition was more effective than either one alone.

**Table 3:** Impact of water irrigation quantity, hydrogel and GB on proline and free amino acids (mg/100 g dry wt.) of peanut plants

WIQ	Hydrogel (kg ha^-1^)	GB (mM)	proline	FAA
			(mg/100 g dry wt)	
100%	0	0	31.86 q	225.15 p
		10	34.56 p	245.63 n
		20	37.41 n	250.13 m
	40	0	36.58 o	237.07 o
		10	39.64 m	244.79 n
		20	43.40 k	257.63 m
	80	0	40.76 l	250.13 l
		10	45.08 j	264.24 k
		20	49.91 h	278.13 j
75%	0	0	45.96i	286.99i
		10	50.71 g	297.02 h
		20	56.97 e	310.99 f
	40	0	50.84 g	307.51 g
		10	56.19 f	319.93 e
		20	61.57c	329.12 d
	80	0	60.52d	347.16 c
		10	66.34 b	355.48 b
		20	74.41 a	369.03 a

Mean values within the same column for each trait with the same lower-case letter are not significantly different at *p* ≤ 0.05

### Changes in growth traits

According to the data in (Tables 4) and 75% WIQ water irrigation significantly reduced *p* ≤ 0.05 growth parameters of peanut plants, including shoot length (cm), number of branches and leaves per plant, shoot fresh and dry weight (g), and root fresh and dry weight, while significantly increased root length (cm) compared with 100% WIQ (control) plants. While hydrogel addition to soil with (40 and 80 kg ha^− 1^) or foliar treatment of peanut plant with 10 and 20 mM GB caused significant increases *p* ≤ 0.05 in different studied growth parameters as compared with untreated control plants under the two irrigation levels (100% and 75% WIQ). Regarding the interactive effect of different concentrations of hydrogel added to soil (40 and 80 kg ha^− 1^) and foliar treatment of 10 and 20 mM GB on peanut plant under the two water levels, Furthermore, GB treatments caused more marked increases with hydrogel addition with the two used levels not only under normal irrigation (100% WIQ) but also alleviated drought stress effect compared with their corresponding controls. Data clearly show the highest increases in different growth traits were obtained by GB treatment with 20 mM under normal and stressed conditions. Herein, the most effective treatment is 80 kg ha^− 1^ hydrogel with 20 mM GB under both water levels as it caused the highest increases of the most studied growth parameters (Table 4).

**Table 4:** Impact of irrigation quantity, hydrogel and GB and their interaction on growth parameters of peanut. Data are means of two seasons

WIQ	Hydrogel (kg ha^− 1^)	GB mM	shoot length (cm)	branches number/ plant	leaves number/ plant	root length (cm)	shoot fresh weight (g)	root fresh weight (g)	shoot dry weight (g)	root dry weight (g)
100%	0	0	23.00f-i	11.00b-e	62.00ij	11.00 h	55.6-fg	1.79f	24.40e	1.79d-g
		10	24.00e-g	12.33 ab	76.33 g-i	12.67 fg	73.13 df	2.09ef	32.53b-d	2.42a-e
		20	25.00d-g	13.00 ab	90.33eg	12.67 fg	88.95b-d	3.68a-d	34.28b-d	2.40a-e
	40	0	26.00c-e	12.00a-c	90.00e-g	11.67gh	63.90ef	2.30 d-f	30.49 cd	2.07b-f
		10	27.33b-d	12.67 ab	107.33 cd	13.33ef	86.29b-d	2.62c-f	23.29 e	2.43a-e
		20	29.00 b	12.67 ab	121.33a-c	13.33ef	98.22a-c	2.71c-f	33.92b-d	3.04 a
	80	0	27.67bc	13.00 ab	97.00 d-f	13.00e-g	81.49c-e	2.85b-f	31.08b-d	2.61a-d
		10	29.00 b	13.33 a	123.67 ab	13.67 de-	104.77 ab	3.67a-d	36.81 ab	2.49a-e
		20	32.67 a	13.00 ab	134.00 a	14.00c-f	108.83 a	3.99abc	41.71 a	2.74a-c
75%	0	0	21.33 hi	9.33 e	51.00 j	11.67gh	41.31 g	2.24ef	14.11 f	0.63 h
		10	22.67 g-i	10.00 de	67.00i	14.00c-f	43.64 g	3.26a-e	19.97 e	0.98gh
		20	23.67e-h	11.00b-e	73.67 hi	15.00a-d	69.83 d-f	4.21 ab	25.07 e	1.64efg
	40	0	21.00i	9.67 e	74.00 hi	13.33ef	55.69 fg	2.99b-f	20.67 e	1.29f-h
		10	22.67 g-i	11.00b-e	85.00f-h	14.33b-e	73.62 d-f	4.16 ab	30.29 d	1.61e-g
		20	25.33c-f	12.00a-c	115.33bc	15.67 ab	88.60b-d	4.54 a	34.08b-d	2.38a-e
	80	0	24.00e-g	10.33c-e	83.33f-h	15.33a-c	75.75 de	3.95a-c	22.77 e	1.35f-h
		10	26.00c-e	11.67a-d	100.33 de	15.67 ab	86.45b-d	4.22 ab	33.39b-d	2.03c-f
		20	24.67e-g	12.00a-c	107.33 cd	16.00 a	89.57b-d	4.53 a	36.17bc	2.97 ab

Mean values within the same column for each trait with the same lower-case letter are not significantly different at *p* ≤ 0.05

### Changes in seed yield and its components

The obtained data of the effect of hydrogel soil addition with 40 and 80 kg ha^-1^ and foliar treatment of 10 and 20 mM or GB to peanut plants grown under different water irrigation quantities are presented in (Table 5). Data clearly show that decreasing irrigation water to 75% WIQ significantly decreased plant weight (g), pod number per plant, seed yield per plant (g), 100 seeds weight (g) and seed yield (ton ha^-1^), straw yield (ton ha^-1^) and biological yield (ton ha^-1^) as compared with those plants irrigated with 100% WIQ. Meanwhile, hydrogel polymer addition to soil and foliar treatment of GB caused significant increases *p* ≤ 0.05 in the above-mentioned yield parameters of peanut plant compared with control plants.

Moreover, all yield attributes of peanut plants markedly responded to the combination of irrigation levels, hydrogel soil amendment, and GB foliar application (Table 6). As expected, the values of seed weight (g), pods number per plant, seeds yield per plant (g), 100 seeds weight (g) and seed yield (ton ha^-1^), straw yield (ton ha^-1^) and biological yield (ton ha^-1^) significantly increased with different levels of hydrogel soil amendment and/or GB treatments compared with untreated controls under both irrigation levels (normal 100% and stressed 75% WIQ). Moreover, higher level of hydrogel or GB was more effective than lower ones (Table 5). Also, the effect of hydrogel with different levels in combination with GB with two levels were more effective than each of them lonely as it caused more marked increases in different studied seed yields and its components in both irrigation levels. In this respect, the combination of 100% WIQ in conjunction with hydrogel polymer (80 kg ha^-1^) with 20 mM GB showed the highest values of seed yield and pod yield of peanut plant compared with other treatments. 80 kgh^-1^ + 20 mM GB caused the greatest increases in different yield parameters it increased seed yield (ton ha^-1^) and biological yield (ton ha^-1^) under 100% WIQ by 72.45% and 78.92% and under 75% WIQ by 141.37% and 112.97% respectively.

**Table 5:** Impact of water irrigation quantity, hydrogel and GB on yield and its components of peanut plants. Data are means of two seasons

WIQ%	Hydrogel (kg ha^− 1^)	GB (mM)	plant weight (g).	pods number/plant.	seed yield/plant (g)	100 seed weight (g)	seed yield (ton/ha)	straw yield (ton/ha)	biological yield (ton/ha)
100%	0	0	140.92gh	29.33ij	31.03 gh	29.65 fg	2.363gh	7.459ef	9.822gh
		10	162.30 fg	48.67 f	52.28 ab	38.00 b	3.981 ab	7.483ef	11.464 fg
		20	193.95 cd	61.67a-c	58.68 a	43.30 a	4.469 a	9.426c-e	13.895 cd
	40	0	182.32c-f	50.00ef	41.40 c-e	33.88 cd	3.153cde	9.849 cd	13.002c-f
		10	194.85 cd	55.33 de	44.13 cd	37.04bc	3.361 cd	10.604b-d	13.965 cd
		20	198.66b-d	64.67 a	48.85 bc	36.37bc	3.720bc	10.537b-d	14.257b-d
	80	0	202.51bc	57.33 cd	46.44 b-d	33.25 cd	3.537b-d	11.016b-d	14.553bc
		10	222.28 ab	63.33 ab	53.50 ab	30.88 d-f	4.035 ab	11.997 ab	16.071 ab
		20	241.83 a	66.33 a	52.99 ab	33.66 cd	4.075ab	13.537 a	17.573 a
75%	0	0	101.96i	25.33 j	19.23 i	18.39 j	1.465 i	5.366 g	6.831i
		10	127.72 h	39.00gh	35.75 e-g	21.69ij	2.723eg	6.086 fg	8.809 h
		20	181.24c-f	47.67 f	40.49 d-f	27.26f-h	3.084 d-f	9.835 cd	12.919c-f
	40	0	158.08 fg	34.33 hi	27.11 h	22.00ij	2.065 h	9.076c-e	11.140 fg
		10	167.32ef	41.00 g	38.43 d-g	26.46gh	2.927d-g	8.923 de	11.850ef
		20	176.67 d-f	46.67 f	43.69 c-e	27.11f-h	3.327cde	9.241c-e	12.568 d-f
	80	0	189.92c-e	41.33 g	32.40f-h	25.30 hi	2.468f-h	11.118bc	13.586c-e
		10	194.16 cd	50.67ef	39.04 d-g	29.32e-g	2.973d-fg	10.938b-d	13.911 cd
		20	202.42bc	58.33b-d	46.43 b-d	28.45f-h	3.536b-d	11.010b-d	14.546bc

Mean values within the same column for each trait with the same lower-case letter are not significantly different at *p* ≤ 0.05

### Changes in nutrient contents:

**Fig. 3:**
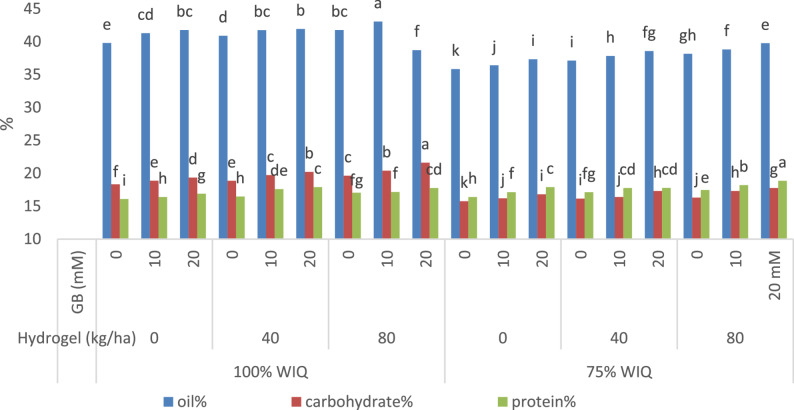
Mean values within the same column for each trait with the same lower-case letter are not significantly different at p ≤ 0.05. Impact of water irrigation quantity, hydrogel and GB on nutrient content (oil%, carbohydrate% and protein%), of peanut plants.

 Concerning the yield quality, namely oil%, carbohydrates% and protein% (Fig. 3) in addition to oil yield ton/ha and protein yield (kg/ha) (Table 6) of peanut yielded seeds, it is obvious that there is a significant decline (*p* ≤ 0.05) in oil% and carbohydrates% while there is a significant increase in protein% of peanut seeds (Fig. 3) at WIQ 75% compared with 100% WIQ irrigation level. Meanwhile, hydrogel with 40 and 80 kg ha^− 1^ or foliar treatment with 10 and 20 mM GB significantly increased the above-mentioned seeds nutrient contents compared with control plants without hydrogel soil addition and GB treatments. Regarding the interactive effect of different treatments (hydrogel and GB) and irrigation water levels (100% and 75% WIQ), data presented in (Fig. 3); (Table 6) show that hydrogel different levels + GB foliar treatments significantly increased oil%, carbohydrate% and protein% of the yielded peanut seeds under 100% WIQ. Furthermore, those treatments alleviated the reduced effect of decreasing WIQ (75%) by improving the above mentioned parameters (Fig. 3); (Table 6). Furthermore, the interaction effect of GB and hydrogel treatments caused more significant increases in the above mentioned parameters. Data clearly show the superiority of 20 mM GB and 80 kg ha^− 1^ hydrogel over the other treatments under different irrigation levels.

**Table 6:** Impact of water irrigation quantity, hydrogel and GB on nutrient content (oil yield (ton ha^-1^) and protein yield (kg ha^-1^) of peanut plants

WIQ	Hydrogel (kg ha^− 1^ )	GB (mM)	oil yield (ton/ha^− 1^)	protein yield(kg/ha)
100%	0	0	0.941 fg	380.25gh
		10	1.644 a-c	652.55a-c
		20	1.867 a	754.87 a
	40	0	1.290 de	518.99 d-f
		10	1.403 cd	591.48b-d
		20	1.559 bc	665.91 ab
	80	0	1.477 cd	602.99b-d
		10	1.754 ab	699.19 ab
		20	1.562 bc	716.70 a
75%	0	0	0.525 h	240.08i
		10	0.991 fg	466.13e-g
		20	1.151 e.g.	552.03c-e
	40	0	0.766 g	353.27 h
		10	1.107 ef	519.85 d-f
		20	1.283 de	591.28b-d
	80	0	0.941 fg	430.59f-h
		10	1.154 ef	541.09c-e
		20	1.406 cd	666.56 ab

Mean values within the same column for each trait with the same lower-case letter are not significantly different at *p* ≤ 0.05

### Water productivity (WP)

Irrigation of peanut plants with 75% WIR has no effect in water productivity compared with unstressed plants (100% WIR) (Table 7). Meanwhile, treatment of plants with different levels of hydrogel soil amendment by (40 and 80 kg ha^− 1^) or GB (10 and 20 mM) treatments increased significantly water productivity compared with untreated control plants. Regarding the interactive effect of hydrogel soil amendment of (40 and 80 kg ha^− 1^) and GB foliar treatment, under different water irrigation quantity (100% and 75% WIR), (Table 7) showed that, both of hydrogel and GB increased markedly WP in peanut plants grown under either well-watered or water stressed conditions. The higher concentration used 20 mM of GB, followed by 10 mM, recorded the highest values of WP in peanut plants subjected to 75% and 100% WIR, respectively, compared with control treatments, the addition of hydrogel as soil amendment by (40 and 80 kg ha^− 1^) and/or GB (10 and 20 mM) as foliar treatments under water stress increased peanut productivity and saving 25% from irrigation water consumption compare with the control of 100% well-watered (Table 7).

**Table 7:** Effect of hydrogel and GB on water productivity (WP) of peanut grown under different water irrigation quantity (WIQ)

WIQ	Hydrogel (kg ha^− 1^)	GB (mM)	WP kg seeds m^−3^water ha^− 1^
100%	0	0	0.496 hi
		10	0.836 b-e
		20	0.939 ab
	40	0	0.662 fg
		10	0.706 d-g
		20	0.782 c-f
	80	0	0.743 c-f
		10	0.856 a-d
		20	0.848 a-d
75%	0	0	0.410 i
		10	0.763 c-f
		20	0.864 a-c
	40	0	0.578 gh
		10	0.820b-e
		20	0.932 ab
	80	0	0.691 e-g
		10	0.833 b-e
		20	0.991 a

Mean values within the same column for each trait with the same lower-case letter are not significantly different at *p* ≤ 0.05

### Pearson correlation coefficient (PCC) heat map matrix, with significance levels

**Fig. 4:**
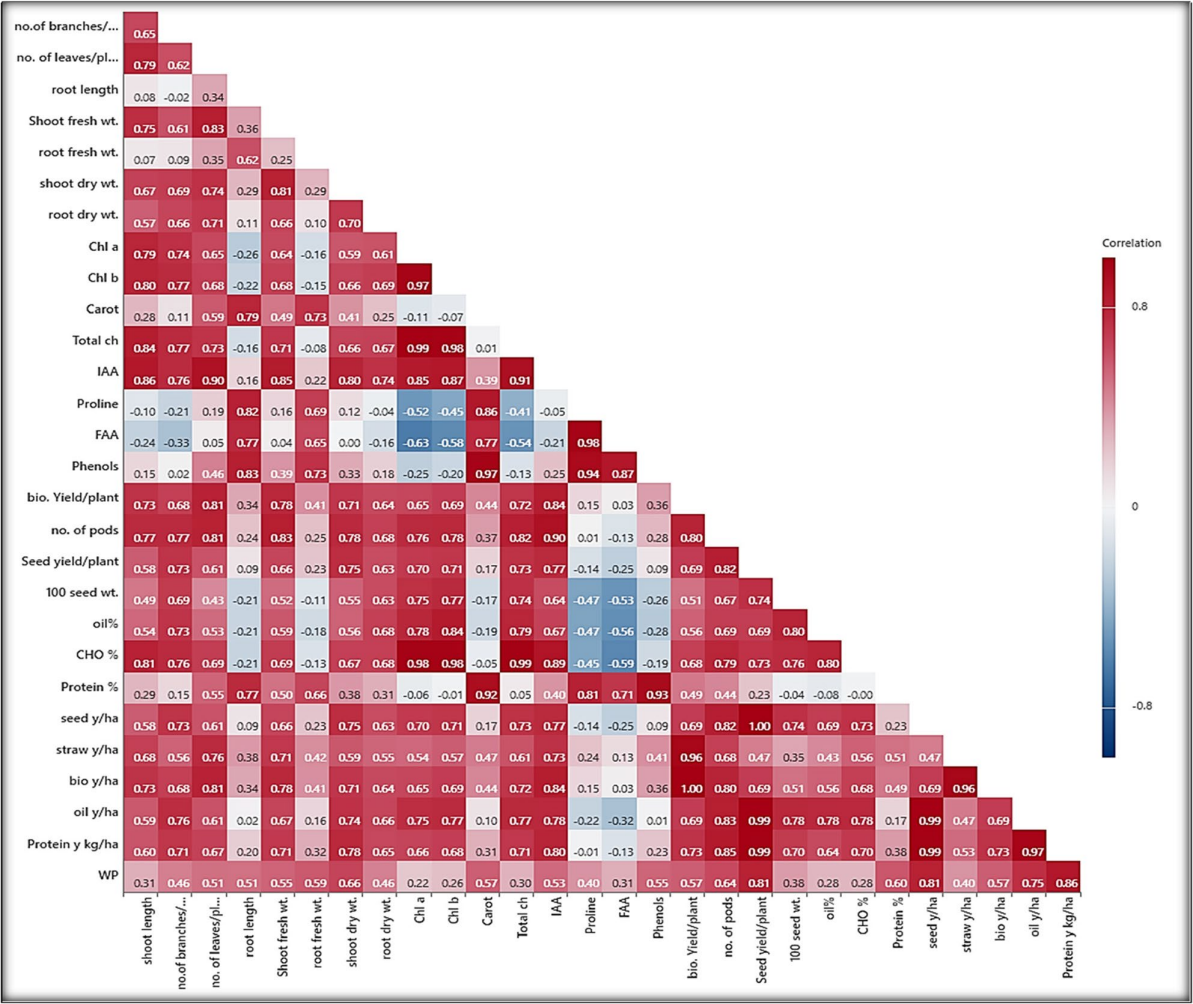
With significance levels (* p < .05, ** p < .01, *** p < .001). Red and Blue color represent positive and negative correlations, respectively Heat map of Pearson correlation analysis of all investigated traits of hydrogel soil applications and foliar application of glycinebetaine under two water irrigations levels on growth, yield and its components as well as seed quality of peanut under sandy soil conditions

Figure 4 Represents the correlation coefficients between various traits and variables related to the peanut plants. Correlation coefficients range from − 1 to 1, with − 1 indicating a strong negative correlation, 1 indicating a strong positive correlation, and 0 indicating no correlation. The summary of the correlations was, shoot length has positive correlations with all other variables, such as number of branches/plant, no. of leaves/plant, root length, shoot fresh wt., root fresh wt., shoot dry wt., root dry wt., Chlo a, Chlo b, carotenoids, total chlorophyll, IAA, Phenols, biological yield/plant, number of pods/plant, seed yield/plant, 100 seed wt., oil%, CHO %, protein %, seed yield (ton ha^− 1^), straw yield (ton ha^− 1^), biological yield (ton ha^− 1^), oil yield (ton ha^− 1^), protein yield (kg ha^− 1^), and water productivity. This suggests that, shoot length has some influence on these traits. Number of branches plant^− 1^ it is has positive correlations with several variables, including number of leaves/plant, shoot fresh wt., root fresh wt., shoot dry wt., root dry wt., Chlo a, Chlo b, carotenoids, total chlorophyll, IAA, Phenols, biological yield/plant, no. of pods/plant, seed yield/plant, 100 seed wt., oil%, CHO %, protein %, seed yield (ton ha^− 1^), straw yield (ton ha^− 1^), biological yield (ton ha^− 1^), oil yield (ton ha^− 1^), protein yield (kg ha^− 1^), water productivity. It may play a role in the growth and development of the peanut plant, number of leaves/plants has strong positive correlations with all other studied traits. This suggests that no. of leaves/plant is closely related to these variables. Root length also shows strong positive correlations with shoot fresh wt., root fresh wt., shoot dry wt., root dry wt., carotenoids, IAA, proline, FAA, phenols, bio. yield/ plant, no. of pods/plant, seed yield plant^− 1^, protein %, seed yield (ton ha^− 1^), straw yield (ton ha^− 1^), biological yield (ton ha^− 1^), oil yield (ton ha^− 1^), protein yield kg ha^− 1^ and water productivity, these means that the root length, may be influenced by these traits. Shoot fresh weight also shows strong positive correlations with all other studied variables. This suggests that shoot fresh wt. has some influence on these all-studied traits. Root fresh wt. has positive correlations with shoot dry wt., root dry wt., carotenoids, IAA, proline, FAA, Phenols, bio. yield plant^− 1^, no. of pods plant^− 1^, seed yield plant^− 1^, protein %, seed yield (ton ha^− 1^), straw yield (ton ha^− 1^), biological yield (ton ha^− 1^), oil yield (ton ha^− 1^), protein yield kg ha^− 1^, water productivity, this means that it may play a role in the growth and development of the peanut plant. Shoot dry wt. also shows strong positive correlations with all other studied variables. This suggests that shoot dry wt. has some influence on these all-studied traits. Chlo a and Chlo b shows strong positive correlations with total chlorophyll, IAA, bio. yield plant^− 1^, no. of pods plant^− 1^, seed yield plant^− 1^, 100 seed wt., oil%, CHO %, seed yield (ton ha^− 1^), straw yield (ton ha^− 1^), biological yield (ton ha^− 1^), oil yield (ton ha^− 1^), protein yield kg ha^− 1^, and water productivity. Carotenoids shows strong positive correlations with total chlorophyll, IAA, proline, FAA, Phenols, biological yield plant^− 1^, number of pods plant^− 1^, seed yield plant^− 1^, protein %, seed yield ha^− 1^, straw yield seed yield (ton ha^− 1^), straw yield (ton ha^− 1^), biological yield (ton ha^− 1^), oil yield (ton ha^− 1^), protein yield kg ha^− 1^, and water productivity. Biological yield plant^− 1^, number of pods plant^− 1^, seed yield plant^− 1^, 100 seed wt., oil%, CHO %, protein %, seed yield seed yield (ton ha^− 1^), straw yield (ton ha^− 1^), biological yield (ton ha^− 1^), oil yield (ton ha^− 1^), protein yield kg ha^− 1^, and water productivity appears to be related with several studied variables. Proline, FAA, and phenols show a strong negative correlation with the traits of seed yield plant^− 1^, 100 seed wt., oil%, CHO %, seed yield ton ha^− 1^, oil yield ton ha^− 1^, protein yield kg ha^− 1^. 

## Discussion

### Growth parameters

Water scarcity is a significant issue in agriculture that hinders plant growth and development and lowers agricultural productivity. Peanuts, as an oilseed crop, benefit from enhanced soil moisture, as well as optimised nutrition and photosynthetic transport facilitated by the use of hydrogel, peanuts gain from increased soil moisture as well as improved nutrition and photosynthetic transport made possible by hydrogel [54]. The current investigation found that a water deficit considerably lowered the growth criterion of peanut plant (Table 3). These reductions may be caused by decreased cell turgor pressure, which prevents cell division and expansion and ultimately restricts overall growth [55]. Also, the decreased the uptake of nutrients such as nitrogen, phosphorus, and potassium, photosynthesis, and energy input, can be linked to decreased plant height, number of branches and leaves per plant [56, 57]. Consequently, these responses accounted for the reduced accumulation of dry matter in both the shoot and root. The decreases caused by water scarcity align with the findings of Rady [58], Sadak [59], Bakry [60], El-Bassiouny [61] and Al-Ashkar [62]. While the increased root length is consistent with the results of Gámez [63], who found that plants under water stress exhibited greater root length than control plants. It is also possible that peanut cells began to redirect assimilations from the stem to encourage root growth and higher water absorption [64]. However, the use of hydrogel ameliorated drought-induced damages, given that hydrogel improved peanut growth parameters by enhancing soil moisture-retaining along with its subsequent gradual release over extended periods, enables the plant to utilise root zone hydration more efficiently under reduced watering conditions [65, 66]. Furthermore, applying hydrogel promotes plant vitality, especially in environments with limited moisture, boosts the production of dry matter, and prolongs the stay-green quality [67]. Moreover, Rezashateri [68] and Liao [69] reported that hydrogels seemed to increase root growth, since the formation of a better root system is favourable to obtain efficient utilization of water and nutrient resources. Moreover, hydrogel application enhanced the uptake of micro- and macro-nutrients, particularly nitrogen and potassium, which contributed to increased plant dry weight [70, 71]. Furthermore, GB treatment could improve plant growth of peanut plant under normal or drought stressed conditions. The following possible tactics have been approved for use in conjunction with GB treatment’s stress reduction: (1) maintaining water status [72] (2) a decrease in ABA and an increase in growth promoters (IAA, GAs, salicylic acid, and cytokinin) [73], (3) a rise in cell division and enlargement as a result of activated water absorption and elevated P content [74]. Furthermore, according to Annunziata [31], Sharma and Kaushik [75]; Khoshkharam [76] and Abd Elhamid [72], the exogenous application of GB could mitigate the negative effects of drought by enhancing the vigor of root and shoot growth, leaf area, pigment content retention, raising the concentration of osmoprotectants, preventing the concentration of polysaccharides, and/or stabilising vital proteins, finally plant yield.

### Photosynthetic pigments

Photosynthetic productivity a crucial physiological indicator, has been used extensively to assess the vigor of plant growth. This study found that stressed peanut plants reduced Chlo a, Chlo b, and total pigment contents, which is thought to be a sign of chlorophyll degradation caused by ROS damage [59, 77]. The overproduction of ROS due to drought stress inhibited photosynthetic electron transport chain and hindered the manufacture of many constituents of photosynthetic pigments [78]. Additionally, this diminished effect results from diffusion limitation brought on by stomata closure and decreased Rubisco content that may impact CO_2_ assimilation rates [79]. Our obtained data are in accordance with those obtained by Sadak [80] and Abd Elhamid [81]. Plants cultivated with hydrogel exhibit increases in photosynthetic pigments as compared to control plants (Fig. 1). The ability of the environmentally friendly hydrogel polymer to either reduce chlorophyll degradation or increase chlorophyll biosynthesis by giving the plant an adequate supply of water and nutrients when it is experiencing a water deficit condition may be the cause of the increases in different components of photosynthetic pigments [82]. In a number of crops, including tomatoes [83] and canola [84], hydrogel has been demonstrated to promote photosynthetic pigment under stressful conditions. Additionally, because GB prevents photoinhibition [85], protects the lipids and Rubisco enzyme of the photosynthetic apparatus, and preserves electron flow through thylakoid membranes, which in turn maintains photosynthetic efficiency [86], it may have a positive effect on peanut photosynthetic pigments by lessening the negative effects of water deficit conditions on plants’ ability to photosynthesize.

### Endogenous IAA and phenol contents

Regarding the negative effect of water deficit on IAA in peanut leaves, this decrease might be due to a higher rate of IAA breakdown or its conversion to an inactive state. Additionally, according to González-Villagra [87], water deficit disrupts the production of endogenous phytohormones by increasing ABA concentration, which lowers IAA. According to Kazan [88], these decreases in IAA might be attributed to a transcription factor that is sensitive to stress and controls auxin and root growth. However, the addition of hydrogel to soil could ameliorate this negative impact by increase IAA. These increases might be due to decreased IAA degradation and reduced IAA oxidase activity [84]. Furthermore, GB may have a promotional effect on peanut plant IAA by accelerating growth promoters (IAA, GA3, and cytokinins), which increases drought tolerance, while also reducing the accumulation of inhibitors, which are represented by ABA plants [72].

Meanwhile, phenol content was increased in peanut leaves. This increases has the potential to lessen the negative effects of drought stress [89]. Specifically, in plants under drought stress, the increase in reactive oxygen species levels is often correlated with modifications in net carbon acquisition that have a major effect on secondary organic compound signaling pathways, particularly leaf polyphenols [90]. The earlier obtained results of Elewa [91] on quinoa and Ezzo [92] on Moringa, confirmed our results of decreasing IAA and increasing phenol contents. Additionally, hydrogel treatment increased phenols content of peanut plants. Under drought stress, phenols—known as antioxidants—are involved in cellular signaling activities and are generated from a variety of secondary metabolites in the shikimic acid cycle [93, 94]. Total phenol concentration, an antioxidant marker, was higher in tomato plants treated with super water absorbance hydrogel polymer than in the control, indicating increased antioxidative potential [95].

### Osmoprotectants

 Abiotic stress causes plants to sense a disruption in their physiological processes and react quickly by accumulating a variety of osmolytes, primarily proline [96]. In the present study, peanuts showed increases in the concentrations of osmolytes, such as proline and free amino acids [97]. According to Mirfattahi [98], proline increases might caused by decreases in proline oxidase and proline catabolizing enzymes. According to Yang [99], proline plays important roles in osmotic adjustment, stabilization, and the defense of membranes, proteins, and enzymes against the harmful effects of water deficit-induced osmotic stressors. Proline’s ability to directly function as a ROS scavenger and cellular redox status regulator is also clear [100]. With respect to the impact of hydrogel on osmoprotectants namely proline, hydrogel treatment increased the content of proline in leaves compared to control plants. The positive impact of hydrogel treatment on osmotic protector content maybe linked to its role as a soil reservoir [101]. This reduces nutrient loss from the root zone, improves fertilizer use efficiency, stops nutrient leaching, and increases crop water absorption capacity and water use efficiency, particularly on sandy soils [71]. Furthermore, our data unequivocally demonstrate that, in comparison to corresponding controls, GB treatments markedly raised proline concentrations in peanut leaves under drought stress and in unstressed plants. Moreover, Dawood [102] mentioned that GB treatments caused significant increases in phenolic compound, and proline of chickpea plants irrigated with either tap water or saline solution relative to corresponding control. It was reported by Estaji [103] that, phenolic compounds constitute a part of cellular solutes that reduce the environmental stress on the plant because of protecting cells from potential oxidative damage and increasing stability of cell membrane. In addition, glycinebetaine was able to scavenge free radicals, which is more significant than their function as an osmolyte alone.

### Yield and its components

 Water deficit stress decreased yield attributes such as seed weight, pods number per plant, seeds yield per plant, 100 seeds weight and seed yield, straw yield and biological yield of peanuts plant. The main causes of these decreases of might be the decreases in growth criteria, photosynthetic pigments, and photosynthetic outputs. Also, these decreases could be caused by the decreases in branch number and leaf size, which would reduce biomass production, hinder the movement of photo assimilate to the developing seeds, and/or cause flower and pod abortion. The timing, duration, and intensity of drought stress determine how severe the water deficit is for peanut plants [104]. It was discovered that peanut production varied under drought stress at many developmental stages, including as flowering, peg start, and seed maturity. Furthermore, water deficit making soil dry out, causes peg penetration for pod formation to be difficult. Even when the pegs penetrate the soil, reduced water content in the root zone may hinder pod formation leading to decreased seed yield [105]. Moreover, the total carbohydrate, protein, and oil contents of the produced peanut seeds significantly decreased due to drought stress. In addition, the declines in growth parameters and photosynthetic pigments are the primary causes of these decreases in carbohydrate levels. Because they are directly related to physiological functions like photosynthesis, translocation, and respiration, fluctuations in the amount of carbohydrates [106]. Moreover, Oil%, carbohydrate%, protein%, and oil yield kg h^− 1^ of seed yield were all reduced by drought stress (Table 6). Those decreased might be due to reduced chlorophyll content in leaves causing less photosynthetic activity and consequently less carbohydrate buildup in mature leaves, which could lessen the amount of carbohydrates transported from the leaf to the growing seeds [107, 108]. Since carbohydrate alterations are connected to several physiological processes, including respiration, motility, and photosynthesis, they are especially significant when it comes to changes in carbohydrate contents. In addition, the declines in growth parameters and photosynthetic pigments are the primary causes of these decreases in carbohydrate levels. Because they are directly related to physiological functions like photosynthesis, translocation, and respiration, fluctuations in the amount of carbohydrates in the generated [106]. While, low irrigation levels may cause some polyunsaturated fatty acids to oxidase, resulting in lower oil levels in peanut seeds [109]. However, the current study found that using hydrogels improved the yield properties of peanuts. The superiority of hydrogel in improving yield and yield characteristics may be attributed to its function as a soil reservoir, which lowers nutrient loss from the root zone in sandy soil and maximizes plant water uptake efficiency while increasing soil water-holding, water use, fertilizer use, and nutrient leaching prevention efficiency [110]. This data shows that hydrogel may effectively hold and release water over time, increasing the amount of moisture available in the soil for plant absorption. By increasing the number of branches, enhancing plant canopy structure, and having higher chlorophyll content, hydrogel has been shown to improve numerous important elements of peanuts growth, development, and yield. According to Riad [18], hydrogel has a superior effect on increasing sunflower and wheat yield and yield characteristics in Egyptian soils. According to research by Badr [84], adding 40 kg/ha of hydrogel to sandy soil significantly enhanced the canola plant’s growth and production characteristics as well as the percentage of oil and carbohydrates in the seeds that were produced. Also, (Tables 6 and 7)demonstrate that, in comparison to matching controls, all GB treatments significantly increased peanut yield, oil, carbohydrate, and protein contents in the seed yield in plants irrigated with 100% or 75% IWQ. It is evident that the most noticeable and successful treatment for reducing the negative effects of drought stress was 20 mM GB. Because GB regulates ion homeostasis and has an osmoprotective effect on photosynthetic machinery, it encourages plant growth and yield under both normal and stressful conditions [86] as well as enhancing CO_2_ uptake in drought-stressed plants, and due to its function in hormone manufacture and transport, such as cytokinins, which may play a part in the movement of photoassimilates [85]. GB impact on peanut yield might be reflected by the preservation of a greater net photosynthetic rate and an improvement in the source–sink relationship [102]. Because of its osmoprotective effects on photosynthetic machinery and regulation of ion homeostasis, GB promotes plant growth and yield [85] along with enhancing drought-affected plant CO_2_ assimilation [102].

## Conclusion

Overall, from this investigation, it was evident that water stress with 75% water irrigation quantities, decreased plant growth, photosynthetic pigments, indole acetic acid, yield and its components as well as nutritional values of yielded seed of oil%, carbohydrates% and protein%. While hydrogel addition to soil or foliar treatment of glycine betaine alone or in combination effectively mitigated the adverse effects of drought stress on growth and yield of peanut plant. In addition, hydrogel and/or glycinnebetaine also promoted phenols, and compatible solute accumulation in leaves (proline and free amino acids), which were mainly increased under water stress. However, both hydrogel and GB caused significant increases in growth and yield of peanut plant under sandy soil under normal irrigation or water deficit stress but more significant increases were obtained when the added both on plant.

In the future, several key research areas should be thoroughly investigated to advance our understanding and application of superabsorbent like hydrogel and osmoprotectant compounds as glycinebetaine in agriculture.

## Data Availability

The authors declare that all data generated or analyzed during this study are included in this published article.
